# A Brief Review of Gel Polymer Electrolytes Using In Situ Polymerization for Lithium-ion Polymer Batteries

**DOI:** 10.3390/polym15040803

**Published:** 2023-02-05

**Authors:** Wookil Chae, Bumsang Kim, Won Sun Ryoo, Taeshik Earmme

**Affiliations:** Department of Chemical Engineering, Hongik University, Seoul 04066, Republic of Korea

**Keywords:** polymer electrolytes, lithium-ion polymer batteries, in situ polymerization

## Abstract

Polymer electrolytes (PEs) have been thoroughly investigated due to their advantages that can prevent severe problems of Li-ion batteries, such as electrolyte leakage, flammability, and lithium dendrite growth to enhance thermal and electrochemical stabilities. Gel polymer electrolytes (GPEs) using in situ polymerization are typically prepared by thermal or UV curing methods by initially impregnating liquid precursors inside the electrode. The in situ method can resolve insufficient interfacial problems between electrode and electrolyte compared with the ex situ method, which could led to a poor cycle performance due to high interfacial resistance. In addition to the abovementioned advantage, it can enhance the form factor of bare cells since the precursor can be injected before polymerization prior to the solidification of the desired shapes. These suggest that gel polymer electrolytes prepared by in situ polymerization are a promising material for lithium-ion batteries.

## 1. Introduction

Lithium-ion batteries (LIBs) are now extensively used everywhere in our lives, from small personal mobile electronics to electric vehicles (EVs) and large energy storage systems (ESSs) [1,2]. The demand for LIBs has consistently increased with the growth of the EV and ESS market, along with the upcoming ubiquitous electronics era [3].

**Figure 1:**
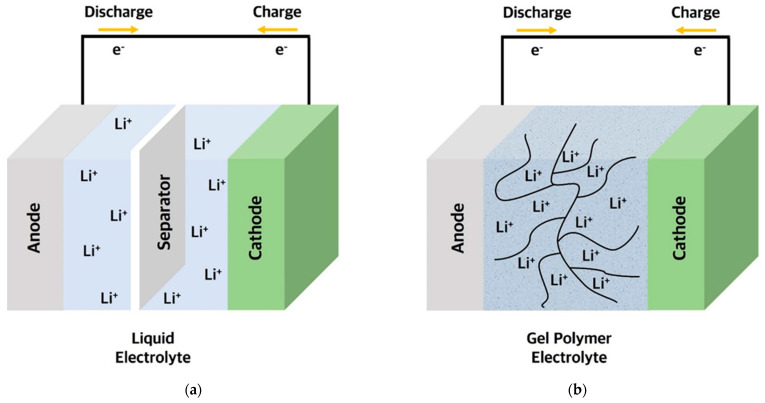
Schematic diagram of lithium batteries using (**a**) liquid electrolyte; and (**b**) gel polymer electrolyte.

LIBs consist of cathode and anode materials with metal foil current collectors, a separator, and an electrolyte. Liquid electrolytes (LEs) have high ionic conductivity (10^−3^–10^−2^ S cm^−1^) and can form a good interfacial contact with the electrode active materials to function as a good pathway for lithium-ion during the charge/discharge cycling process (Figure 1a) [4,5]. The LEs can easily be injected during the manufacturing process of LIBs and permeate into the electrodes and separators. However, leakage and flammability of liquid electrolytes may cause a safety issue of LIBs at abnormal conditions [6,7,8,9]. LEs have a risk that the battery can easily explode or catch fire when the battery cell temperature unexpectedly rises due to the internal short incurred by external impact or formation of lithium dendrite inside. Especially, lithium dendrite is electrically segregated and tends to grow at the anode surface as the battery cycling proceeds. This causes lithium loss with the repeating cycles followed by consumption of excess lithium to replenish it, thus resulting in overall capacity loss. Moreover, the growth of sharp lithium dendrite induces a penetration of the separator that leads to an internal short between the electrodes, resulting in thermal runaway and eventual cell explosion and fire. Additionally, LEs have a narrow electrochemical window that restricts the possibility of using operating voltage above 5 V, which becomes an obstacle to achieving high energy density.

To overcome these issues, research on solid-state electrolytes has been actively conducted to ensure the safety and good electrochemical stability of LIBs. Solid electrolytes can be largely categorized into oxide or sulfide inorganic-based materials or polymer-based electrolytes. Table 1 shows brief advantages and disadvantages of each solid-type electrolyte.

Typical oxide-based inorganic solid electrolyte structures are sodium superionic conductors (NaSICON-type), lithium superionic conductors (LiSICON-type), garnet, and perovskite structures. The representative composition of NaSICON-type is Li_1+x_Al_x_M_2-x_(PO_4_)_3_ (LAMP), and this type typically shows ionic conductivity of 10^−3^–10^−4^ S cm^−1^ and low stability to Li metal. The electrochemical stability of NaSICON-type changes according to property of metal ions. LiSICON-type electrolyte possesses γ–Li_3_PO_4_ structure with low ionic conductivity of 10^−5^ S cm^−1^. However, LiSICON-type demonstrates outstanding stability to bare lithium, which is appropriate to use with Li metal electrode. Li_7_La_3_Zr_2_O_12_ (LLZO) electrolytes with garnet structure have good ionic conductivities (10^−3^–10^−4^ S cm^−1^) despite the possible point contact of LLZO with Li metal electrode. Li_3x_La_2/3−x_□_1/3−2x_TiO_3_ (LLTO) with a composition of ABO_3_ is a perovskite structure that possesses mild ionic conductivity of ~10^−4^ S cm^−1^, although the side effect to Li metal exists. Overall, oxide-based inorganic solid electrolytes possess advantages, such as high ionic conductivity of ~10^−3^ S cm^−1^, good thermal stability, mechanical strength, and air stability. However, they have the disadvantages of high interfacial resistance with electrodes, high resistance between electrolyte particles, undesired reactivity when used with Li metal electrodes, and especially, they require high processing temperatures over 1000 °C, making it difficult to fabricate large area devices [10,11,12,13,14,15,16,17,18].

Sulfide-based solid electrolytes can be categorized into LPS, argyrodite, and Li_4-x_Ge_1-x_P_x_S_4_ (LGPS). LGPS shows high ionic conductivity (~10^−2^ S cm^−1^) and low activation energy, whereas sulfide electrolyte of LPS composition has an ionic conductivity of 10^−3^ S cm^−1^. However, LPS and LGPS sulfide solid electrolytes have substantial reactivity with moisture in ambient conditions to form H_2_S. Argyrodite solid electrolytes with good ionic conductivity of 10^−3^ S cm^−1^ indeed form H_2_S when exposed to moisture. Sulfide-based electrolytes derived from oxide-based electrolytes are fabricated by replacing oxygen ion with sulfur ion. The bonding strength between sulfur and lithium ion, which leads to more free-moving lithium ion, is smaller than oxygen and lithium ion because sulfur ion has lower electronegativity compared with oxygen ion. Sulfide-based electrolytes have a large lithium-ion migration tunnel due to the larger ion radius of sulfur than that of oxygen. Sulfide-based electrolytes have high ionic conductivity of ~10^−2^ S cm^−1^ and react to Li-metal because of the thermodynamically advantageous tendency of Li-metal to form H_2_S when exposed to moisture. These problems severely limit the commercial applications of sulfide-based solid electrolytes [13,19,20,21,22,23]. A comprehensive review of oxide and sulfide-based electrolytes can be found elsewhere [24,25].

Polymer-based electrolytes (PEs) are also relatively free from safety issues such as explosions and fire incidents compared with LEs. Most importantly, PEs based on polymers have good processability and flexibility that can easily be applied to battery manufacturing process. While inorganic-based electrolytes typically need high fabrication temperature to form a solid-state electrolyte phase, PEs can be obtained using room temperature fabrication processes. PEs possess good interfacial properties, such as facile compatibility and interfacial contact between electrode and electrolyte, compared with inorganic-based electrolytes. Despite disadvantages of low thermal and mechanical stability compared with inorganic-based electrolytes, PEs have potential as an alternative to LEs for LIBs as well as electrolytes for Li-metal, Li-S, and Li-air batteries with high energy density (Figure 1b) [26,27,28,29,30,31].

Moreover, conventional LEs are not suitable for flexible batteries since LEs can easily have problems of leakage or internal short when batteries are repeatedly bent or folded, whereas PEs have good potential as electrolytes for flexible batteries. PEs with flexibility do not show leakage or internal short even when the batteries are repeatedly taken in motion [32,33,34]. PEs tends to have high energy density, safety, good processability, and interfacial properties. These properties indeed enable large-scale manufacturing of versatile solid-state secondary batteries. Therefore, the effort on development of advanced PEs is essential for next-generation batteries [35,36,37].

In this brief review, various types of polymer electrolytes (PEs) will be briefly summarized, and the preparation method of PEs will be discussed, with a special emphasis on in situ polymerization.

## 2. Polymer Electrolytes (PEs)

In 1973, PEs were first reported, followed by the demonstration of ionic conductive polymer electrolytes composed of PEO and alkaline salt [38,39]. Since then, many studies regarding PEs have been conducted, and they have been demonstrated and reported to show the following advantages: (a) no electrolyte leakage, (b) low flammability, and (c) effective suppression of lithium dendrite growth [40,41,42]. The battery cells with PEs showed improved safety compared with the cells using organic-solvent-based LEs.

PEs with high safety can be classified into solid polymer electrolytes (SPEs), gel polymer electrolytes (GPEs), and composite polymer electrolytes (CPEs) (Table 2). The criteria for selecting polymeric host for PEs are as follows: (a) fast segmental movement of polymer chain, (b) possessing a polar group assisting the dissolution of lithium salts, (c) high molecular weight, (d) good electrochemical stability, (e) low glass transition temperature (T_g_), and (f) high decomposition temperature of polymer chain. Polymeric host materials commonly used in solid-phase electrolytes are poly(ethylene oxide) (PEO), poly(vinylidene fluoride) (PVDF), poly(vinylidene fluoride-co-hexafluoropropylene) (PVDF-HFP), poly(acrylonitrile) (PAN), poly(methyl methacrylate) (PMMA), poly(vinyl chloride) (PVC), poly(propylene carbonate) (PPC), Poly(diallydimethylammonium) chloride (PDADMACl), and Poly(vinylbenzyltrimethylammonium) bis(trifluoromethanesulfonyl) imide (PVBTMATFSI) (Table 3) [43,44,45,46,47,48,49,50,51].

Solid polymer electrolytes (SPEs) consist of lithium salts and polymers. In this solvent-free system, Li^+^ transport mechanism differs from the system with liquid electrolytes (LEs) in which Li^+^ moves by diffusion. Li^+^ transport of SPEs can be explained as the motion of cation species between complexation sites aided by the segmental movement of a polymer chain in the amorphous region. SPEs show good safety because there is no liquid phase organic solvent that may cause leakage or become a source of fire when changed to the vapor phase by high temperature. SPEs can also act as separators which can divide and prevent the hard short between two electrodes, making it possible to remove the separator and utilize the empty space to increase energy density by adding more electrode components. SPEs have good mechanical properties compared with LEs, which are effective in suppressing lithium dendrite. Although SPEs have high thermal stability and mechanical stability, few disadvantages exist, such as poor interfacial contact, compared with other PEs, and low ionic conductivity due to high crystallinity of the polymer at room temperature [40,52,53,54,55,56].

Gel polymer electrolytes (GPEs) were first reported by Feuillade and Perche [57] and are typically composed of lithium salts, polymers, and organic solvent. GPEs that entrap electrolytes in the polymer matrix have diffusivity of liquid and cohesiveness of solid. The Li^+^ transport mechanism of GPEs is mainly diffusion of Li^+^ through LEs entrapped in a polymer matrix. This interesting property allows the polymeric gel to have the significant advantages of good ionic conductivity of liquid electrolytes as well as low volatility and reactivity. However, GPEs contain a liquid phase in contrast to SPEs, which can be vulnerable to the external pressure generated between the anode and cathode. Poor mechanical strength of GPEs can cause damage to GPEs or leakage of entrapped LEs and result in hindrance of large-scale production of LIBs [58,59,60,61,62].

Composite polymer electrolytes (CPEs) have been studied to remedy shortcomings of both low ionic conductivity of SPEs and low mechanical durability of GPEs. Incorporating various materials, such as ionic liquid, nanocomposites, and/or inorganic-filler, into SPEs or GPEs can enhance ionic conductivity. Since the crystallinity of the polymer matrix is reduced by adding filler that interferes with the arrangement of polymer chains, thus increasing the amorphous region for better Li^+^ migration, thermal stability, mechanical durability, and electrochemical stability can be improved. CPEs rely on the characteristics of filler, such as the particle size, porosity, concentration, surface area, and interaction between polymer chains and filler. Although adding inorganic fillers enhances the performance of CPEs, it is still difficult to disperse materials well in the polymer electrolyte matrix [61,63,64,65].

Polymeric ionic liquid electrolytes (PILEs) are composed of polymeric ionic liquids (PILs), lithium salts, and ionic liquids (ILs). PILs, as polymer matrices, have polymer backbones which are composed of repeating units of ILs. PILs and ILs both have advantages, such as high thermal stability, wide electrochemical operating windows, non-flammability, and very low vapor pressure. GPEs using PILs as polymeric matrices and ILs as organic solvents have the advantage not only of the abovementioned GPEs but also that of ILs. These properties of PILEs show potential as better PEs for lithium-based batteries. Recently, the study for PILEs has been actively conducted and achieved remarkable progress, although the ion transport mechanism of PILEs is still unclear [26,66,67,68,69,70,71,72].

## 3. Ex Situ Preparation Method for Gel Polymer Electrolytes

The gel polymer electrolytes with polymeric matrix can be easily prepared by an ex situ method. The polymeric solution is initially prepared by formation of blends and is applied onto electrode or separators before the assembly of LIBs. Solution casting, phase inversion/separation, and electrospinning methods are typically reported for preparing PEs.

The solution cast method is the most common way to form PEs. Polymer and lithium salt in an organic solvent are dissolved to prepare the blended solution and is casted onto the flat substrate followed by drying of the residual solvent media. PEs can be obtained in the form of solid phase after the post drying process [73]. The phase inversion/separation method is the general method to prepare the porous membrane. The polymer is initially dissolved in organic solvent and casted onto the substrate, followed by immersion process of the polymer in nonsolvent liquid, such as water and ethanol, for phase inversion/separation. Then, the organic solvent in the substrate diffuses to the nonsolvent liquid and, consequently, nonsolvent liquid permeates the polymer. After the phase inversion/separation step, the polymer substrate is dried, and the nonsolvent liquid in the polymer evaporates. As a result, membrane pores are formed in the polymer network. The obtained polymer matrix can absorb LEs to obtain GPEs [74]. The electrospinning method is an effective way to fabricate polymeric nanofiber or microfiber. The porosity, pore size, and thickness can be controlled through the electrospinning method. The obtained polymer matrix is later immersed with LEs, and finally, GPEs are obtained [75].

**Figure 2:**
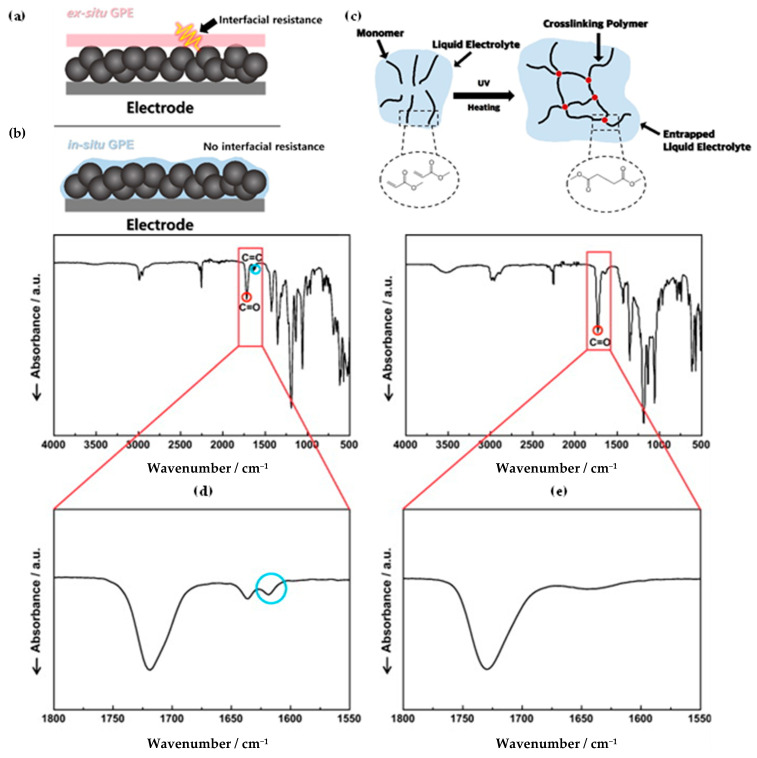
GPEs prepared by (**a**) ex situ and (**b**) in situ; (**c**) in situ polymerization methods; FT-IR spectra of acrylic C=C double bond (**d**) before initiation and (**e**) after initiation. (**d**) Reprinted/adapted with permission from Ref. [78]. Copyright^®^ 2011, Elsevier B. V.

On the contrary, PEs with the ex situ method are typically prepared outside of the cell and later followed by an assembly stage with the anode and cathode. PEs using ex situ methods cannot achieve perfect interfacial contact between anode and cathode, resulting in increased interfacial resistance. Eventually, increased interfacial resistance induces deteriorated cycle performance. [76] (Figure 2a) PEs using ex situ tend to have contact area mostly on the upper part of both electrodes and, thus, have insufficient contact with the deeper part of electrodes. Li^+^ cannot be easily transported to all parts of the electrode due to contact resistance, which causes the capacity loss of the active electrode materials by low utilization. Although insertion of a thin electrolyte film is suggested to decrease interfacial resistance, an additional issue may appear whereby the inserted film is easily damaged and could not sufficiently suppress lithium dendrite [77].

## 4. In Situ Polymerization Method for Gel Polymer Electrolytes

The problems of ex situ processes can be resolved by using in situ polymerization, in which a liquid polymer precursor solution is injected into the electrode and then polymerized by thermal- or photo-curing. The precursor of liquid phase easily immerses into the internal pores of electrode, and finally, PEs can be created inside the pores and on surface of electrode active materials (Figure 2b) [77,79]. The PEs using in situ polymerization can make facile pathway for Li^+^ transport in electrodes and significantly decrease the interfacial resistance due to the good interfacial contact formed between the electrode and polymeric electrolyte [80,81].

The precursor used in in situ polymerization is composed mainly of monomer with functional group, lithium salt, solvent, and initiator. Monomers typically used in in situ polymerization are trimethylolpropane ethoxylate triacrylate (ETPTA), poly(ethylene glycol) diacrylate (PEGDA), trimethylolpropane propoxylate triacrylate (TPPTA), and di(trimethylolpropane) tetraacrylate (DTPTA) (Table 4) [82,83,84,85]. When the radical initiators are activated by thermal- or photo-initiation, the carbo-radicals and/or oxy-radicals attack the C=C bond of acrylate or the C=O bond of aldehyde and ketone, forming a network bridge at the site where the double bond is broken, and consequently, the polymer matrix can be formed (Figure 2c) [86]. The polymerization can be confirmed by FT–IR or Raman spectroscopy to confirm whether PEs through in situ polymerization are well created where the C=C peak in the precursor disappears from GPEs (Figure 2d,e) [78,87,88].

In situ polymerization is commonly initiated by thermal or UV light photo-illumination. The thermal initiation can have an advantage that the thermal energy can be sufficiently transferred to the inside of the cell after the assembly-sealing procedure of the battery cell. However, thermal curing length of at least ~20 to 30 min is required for sufficient polymerization inside the cell which might damage the cell component inside. Furthermore, the relatively longer time will affect the manufacturing lead time, which restricts mass production of batteries. On the other hand, the UV illumination has the advantage of a short preparation time of tens of seconds for full polymerization, though initiation can be possible only before the cell assembly that restricts the facile manufacturing and fabrication conditions. 

### 4.1. Gel Polymer Electrolytes Prepared by Thermal Polymerization

An initiator that can create radicals by thermal treatment, such as benzoyl peroxide (BPO), lauroyl peroxide (LPO), and azobisisobutyronitrile (AIBN), are the typically used initiators. The precursor solution with the thermal initiator was injected into the electrode and thermally cured at 60 to 100 °C depending on the types of initiators [85,86,89,90].

**Figure 3:**
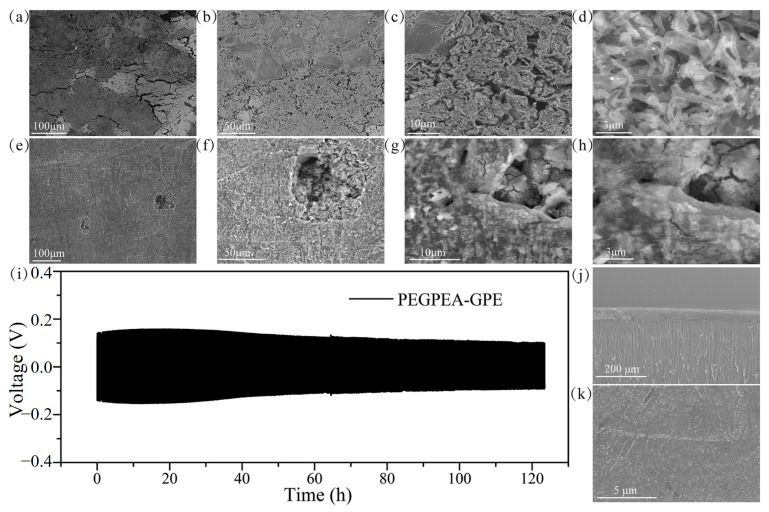
SEM spectra of lithium metal after 70 cycles in 1 M LiPF6-based batteries: (**a**–**d**); PEGPEA-GPE-based batteries (**e**–**h**); (**i**) Voltage profile of Li/PEGPEA-GPE/Li cell at 0.10 mA cm−2; (**j**) Cross-sectional view and (**k**) top view SEM spectra of lithium metal after Li/PEGPEA-GPE/Li cycle test. Reprinted/adapted with permission from Ref. [91]. Copyright^®^ 2018, Elsevier B. V.

PEGPEA–GPEs were prepared using EGPEA, AIBN, and 1.0 M LiPF_6_ in EC/DMC/EMC = 1/1/1 (*v*/*v*/*v*) solvent through thermal curing for 3 h. PEGPEA–GPEs demonstrated high ionic conductivity (3.35 × 10^−3^ S cm^−1^) at 25 °C and wide electrochemical stability of 4.9 V (vs. Li^+^/Li). A rapid mass loss was observed from 323 °C with the TGA measurement that confirmed good thermal stability. Even though PEGPEA–GPEs were rotated, stretched, and tied like rope, PEGPEA-GPEs were still physically not damaged and showed good flexibility. Figure 3 provides SEM images and voltage profiles that demonstrate the lithium dendrite was more effectively suppressed with the PEGPEA-GPEs compared with LEs [91].

**Figure 4:**
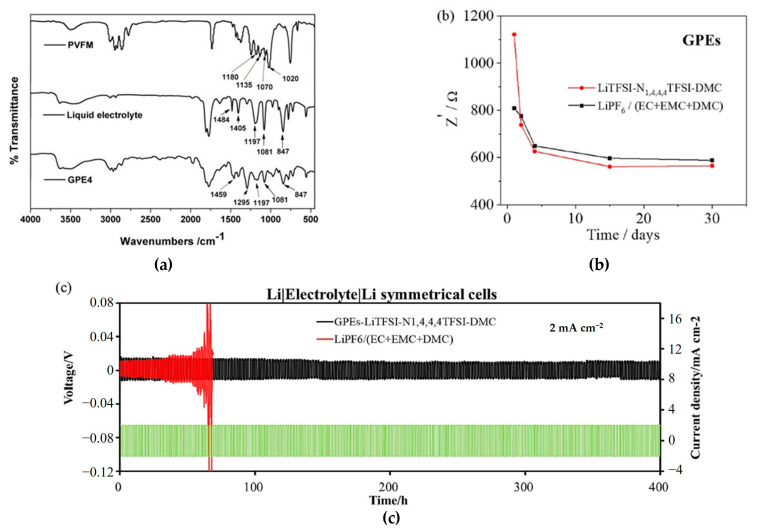
(**a**) FT-IR spectra of PVFM, liquid electrolyte [92], Copyright^®^ 2014, Elsevier B. V.; (**b**) Evolution of the interfacial resistance and (**c**) voltage profiles of Li plating/stripping at a current density of 2 mA cm^−2^. Reprinted/adapted with permission from Ref. [93]. Copyright^®^ 2021, Elsevier B. V.

GPEs having high ionic conductivity (8.82 × 10^−3^ S cm^−1^) were fabricated by thermal polymerization using polyvinyl formal (PVFM) and 1.0 M LiPF_6_ in EC/DMC = 3/7 (*v*/*v*) solvent without the use of an initiator. In Figure 4a, the peaks of 1020, 1070, 1135, and 1180 cm^−1^ indicate that the C-O-C-O-C bond to the ether ring in PVFM completely disappeared in this GPE. In this system, GPEs had interfacial resistance of 64 Ω cm^2^. Compared with interfacial resistance (40 Ω cm^2^) of LEs, GPEs had relatively higher resistance; still, it is noticeable to improve safety despite this slight increase of resistance [92].

GPEs, which have high ionic conductivity of 6.15 × 10^−3^ S cm^−1^ and outstanding Li^+^ transference number of 0.59 at 25 °C, were fabricated using trimethylolpropane trimethacrylate (TMPTMA), lithium bis (trifluoromethane) sulfonimide (LiTFSI), dimethyl carbonate (DMC) with 2,2′-Azobis-(2, 4-dimethylvaleronitrile) (ABVN), and ionic liquids (ILs)-tributylmethylammonium bis(trifluoromethanesulfonyl) imide (N1,4,4,4TFSI) [93]. The GPEs had lower interfacial resistance than LEs over time (Figure 4b). This result demonstrated the suppression of decomposition of passivation layer and uncertain chemical reaction caused by impurities due to the cross-linked structure of ionic liquids, which held and trapped the possible impurities. GPEs did not show any irreversible decomposition below 5.3 V and showed a stable electrochemical window from −0.5 to 5 V. As shown in Figure 4c, GPEs well suppressed lithium dendrite through voltage hysteresis, and the short circuit was not observed in GPEs compared with LEs during the Li plating/stripping process of 400 h. This proves that the GPEs demonstrated here show excellent safety for lithium batteries with good electrochemical stability and suppression of lithium dendrite.

**Figure 5:**
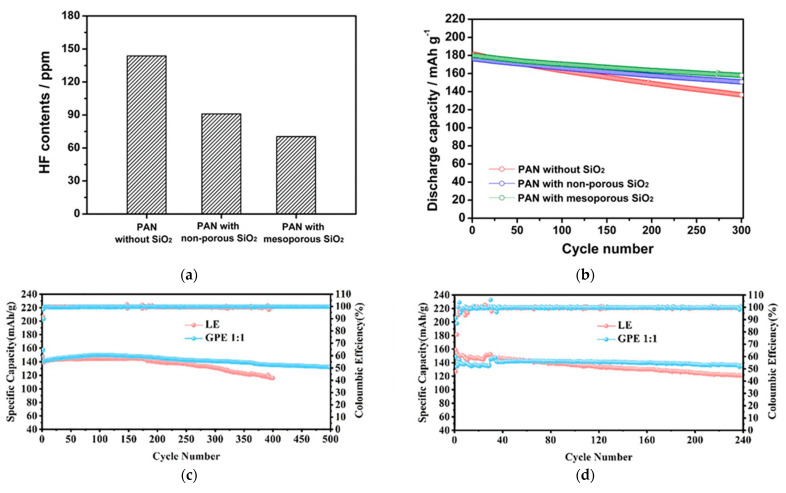
(**a**) HF content in the different electrolytes after being stored at 55 °C for 3 days; (**b**) Discharge capacities of lithium-ion polymer cells assembled with different electrolytes at 25 °C. Reprinted/adapted with permission from Ref. [94]. Copyright^®^ 2016, Nature Publishing Group; (**c**) Cycling performances of LiFePO_4_|LE|Li cell and LiFePO4|GPE 1:1|Li cell with the LiFePO_4_ loading of 1.5 mg cm^−2^ and (**d**) mass loading 3 mg cm^−2^. Reprinted/adapted with permission from Ref. [88]. Copyright^®^ 2022, Elsevier B. V.

CPEs composed of tetra(ethylene glycol) diacrylate (TEGDA), 1.15 M LiPF_6_ in EC/EMC = 3/7 (*v*/*v*), AIBN, and PAN membrane with mesoporous SiO_2_ are reported [94]. The fabricated CPEs had good interfacial adhesion to electrodes and high ionic conductivity (1.80 × 10^−3^ S cm^−1^). Methacrylate-functionalized SiO_2_ (MA-SiO_2_) provided a channel through which Li^+^ could move because of the inner pore due to the mesoporous structure. Since MA-SiO_2_ has a reactive methacrylate group as a cross-linking site, it directly reacted with the precursor to fabricate CPEs. Due to the reactive methacrylate group, the adhesion between the PAN membrane and MA-SiO_2_ became strong, thereby preventing the SiO_2_ particles from detaching from the PAN membrane. CPEs with the abovementioned properties delivered better tensile strength compared with CPEs with non-porous SiO_2_. MA-SiO_2_ also acted as an HF scavenger, in which the prepared CPEs showed reduced HF contents at high temperatures (Figure 5a). The cells using CPEs showed good discharge capacity, equivalent to 88% of initial discharge capacity after 300 cycles at 25 °C compared with other electrolytes (Figure 5b).

Plastic–crystal-embedded elastomer electrolyte (PCEE) composed of succinonitrile (SN) and LiTFSI as plastic crystal, PEGDA and butyl acrylate (BA) as elastomer, and AIBN as thermal initiator are reported [95]. PCEE achieved remarkable mechanical properties, good ionic conductivity (1.10 × 10^−3^ S cm^−1^ at 20 °C), and the high Li^+^ transference number of 0.75. Superior mechanical properties of PCEE were confirmed from tensile and adhesion tests. PCEE showed high extensibility of approximately 300% and high adhesion energy of 21.5 J m^−2^. Generally, PEs have a trade-off relationship between ionic conductivity and mechanical properties, but PCEE did not compromise the relationship. PCEE had remarkable Li reversibility since the stability of PCEE was outstanding, even after 100 cycles at 10 mA cm^−2^ and Li plating/stripping cycle for 1500 h. PCEE maintained a dense and homogeneous surface, resulting from no dendritic and dead lithium.

Poly-DOL GPE composed of lithium difluoro(oxalato)borate (LiDFOB), 1,3–dioxolane (DOL), LiTFSI, and SN were prepared by in situ polymerization [96]. Poly-DOL GPE was prepared without the initiator. Instead, cation-induced ring-opening polymerization of DOL through LiDFOB at appropriate temperature was conducted. The ionic conductivity of poly-DOL GPE by the addition of 30 wt.% SN increased from ~10^−5^ S cm^−1^ to ~10^−4^ S cm^−1^ at room temperature. Poly-DOL GPE showed high anti–oxidation potential above 5 V, while liquid-state DOL electrolyte showed electrochemical decomposition at 4.2 V. The cycling performance was measured at 1 C for 1000 cycles. After the cycling test, capacity of the full cell using pretreated Li metal as anode maintained 105.7 mAh g^−1^, with the superior retention capacity (83.55%), and the coulombic efficiency of cell was ~99%, even after 1000 cycles. On the other hand, capacity of the full cell with unpretreated Li anode showed 79.4 mAh g^−1^, with low-capacity retention of 66.89% and coulombic efficiency of 94.15%.

GPEs with high thermal stability were fabricated by AIBN, 1.0 M LiPF_6_ in EC/DEC/EMC = 1/1/1 (*v*/*v*/*v*) poly(ethylene glycol) dimethacrylate (PEGDMA), and pentaerythritol tetraacrylate (PETEA) through thermal in situ polymerization [88]. Thermal decomposition of GPEs began at 350–450 °C with the PEGDMA and PETEA = 1:1 ratio (GPE 1:1) and achieved good ionic conductivity value of 7.60 × 10^−3^ S cm^−1^. As shown in Figure 5c, the capacity of the cell using GPE 1:1 was 147 mAh g^−1^ after 100 cycles at 0.5 C, and after 500 cycles, GPE 1:1 showed 132 mAh g^−1^, with the capacity retention ratio of 90%. When the mass loading was 3.0 mg cm^−2^, LEs showed higher discharge capacity than GPEs at 60 cycles, presumably due to the good wettability. However, GPEs enabled by in situ polymerization have superior coulombic efficiency and capacity retention of 90% after 60 cycles. (Figure 5d).

In situ polymerization by thermal initiator has an advantage that the thermal energy can be sufficiently transferred into the bare cell even after packing so that the procedure can easily be applied to the manufacturing process. However, relatively longer time can affect lead fabrication time, and the heat treatment can adversely affect inner battery cell components. The polymer electrolytes using thermal polymerization is summarized in Table 5.

### 4.2. Gel Polymer Electrolytes Prepared by UV Photo-Initiation

Polymer matrix can be formed by photo–initiator, which can create initiation radicals when ultraviolet (UV) light is illuminated. The precursor is injected onto and into the electrode, followed by exposure of UV light to incur initiation.

**Figure 6:**
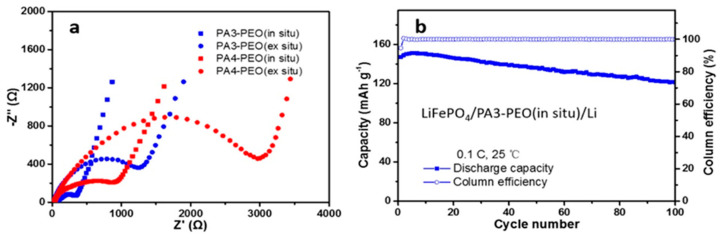
(**a**) Electrochemical impedance spectroscopy of CSPEs; (**b**) Cycling performance of LiFePO4/PA3-PEO/Li cells at 25 °C. Reprinted/adapted with permission from Ref. [97]. Copyright^®^ 2022, American Chemical Society.

Two composite SPEs (CSPEs) using PA3-PEO and PA4-PEO are reported as photo-initiated PE matrix structure [97]. CSPEs were fabricated using ethoxylated trimethylolpropane triacrylate (ETPTA, A3), pentaerythritol tetraacrylate (PETA, A4), PEO, HMPP, LiTFSI, and propylene carbonate (PC) with acetonitrile solvent. CSPEs achieved enhanced ionic conductivity of 2.21 × 10^−5^ S cm^−1^ for PA3-PEO and 7.76 × 10^−6^ S cm^−1^ for PA4-PEO at 25 °C. The PEs were analyzed to have lower crystallinity due to possessing endothermic peak at a lower temperature with A3 and A4 with PEO compared with pure PEO film. PA3/4-PEO resulted in being more stable at high potential. Especially, PA3-PEO showed a stable oxidation potential up to 4.7 V (vs. Li^+^/Li). Li^+^ can be easily transported due to the lower interfacial resistance of CSPEs which was prepared in situ compared to the ex situ method. PA3-PEO CSPEs also showed a good coulombic efficiency of 96% after 100 cycles. (Figure 6a,b)

UV-SPEs with ionic conductivity of 2.95 × 10^−5^ S cm^−1^ and electrochemical stability window of 4.96 V (vs. Li^+^/Li) were studied. UV-SPEs composed of poly(ethylene glycol) methyl ether methacrylate (PEGMEMA), poly(ethylene glycol) diacrylate (PEGDA), and LiTFSI with HMPP were used as an initiator for PE precursor. The fabricated UV-SPEs had initial thermal decomposition (5% weight loss) temperature of polymer at 321.94 °C, which is an outstanding thermal stability. For UV-SPEs paired with a lithium metal electrode, the potential value was stable at approximately 0.06 V at current density of 0.1 mA cm^−2^ and no short circuit appeared up to 800 h. Although the current density became 0.15 mA cm^−2^ at 60 °C, hard short of both electrodes in Li|LEs|Li cell occurred at 220 h, and the Li plating/stripping process lasted more than 250 h with UV-SPEs. This result suggests that the UV-SPEs can well suppress side reaction that might occur with LEs and demonstrates that UV-SPEs are significantly more stable than LEs. The cell using LiMn_x_FE_1-x_PO_4_ (LMFP) as the cathode obtained a maximum discharge capacity of 158.8 mAh g^−1^ at 0.2 C and a discharge capacity of 134.4 mAh g^−1^ after 240 cycles at 60 °C [98].

**Figure 7:**
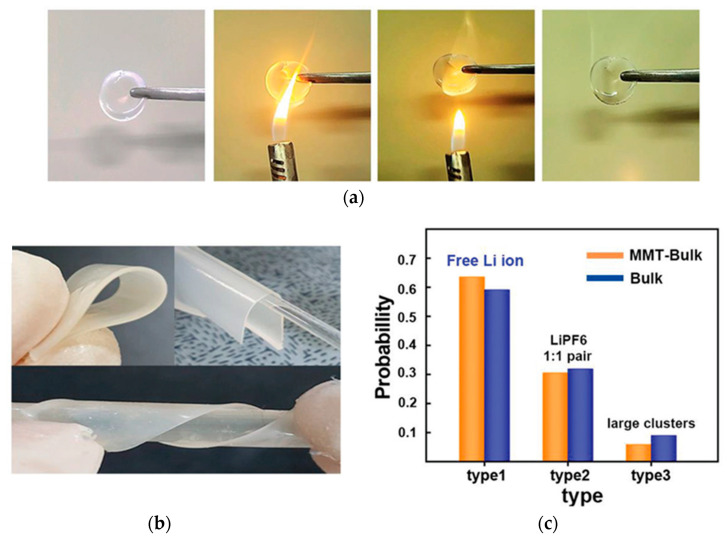
(**a**) Flammability test of the CGPEs; (**b**) Photographs of the Flexibility CPEs; and (**c**) The distribution Li ion clusters. Type 1: Free Li ion, Type 2: One Li-ion PF_6_ pair, Type 3: One Li-two PF_6_ cluster, Two Li-ion PF_6_ cluster, and the others. Reprinted/adapted with permission from Ref. [99]. Copyright^®^ 2020, John Wiley & Sons Inc.

U-CPCE using ETPTA, PVdF-HFP, 1.0 M LiPF_6_ in EC/DEC = 1/1 (*v*/*v*) with HMPP initiator along with the incorporation of montmorillonite (MMT) showed high ionic conductivity (1.60 × 10^−3^ S cm^−1^) and good thermal stability [99]. Lithium dendrite was well suppressed due to the durable mechanical properties of U-CPCE. As shown in Figure 7a,b, remarkable thermal stability and flexibility were achieved, originating from the synergetic effect of the flame-retardant properties of MMT, stable semi-interpenetrating network (IPN) structure, and lasting semi-IPN matrix consisting of crosslinked ETPTA. U-CPCE showed a Li^+^ transference number of 0.78, presumably due to the incorporation of MMT with a high dielectric constant so that Li^+^ could be more easily dissolved into the MMT layer. The polymer matrix interferes with the coordination of Li^+^ and PF_6_^−^ to have freer Li^+^ (Figure 7c) by trapped PF_6_^−^.

CPEs were also prepared via solvent-free in situ polymerization using acrylate functionalized poly-ε-caprolactone (PCLA), lithium bis(flourosulfonyl)imide (LiFSI), halloysite nanotubes (HNTs), and 4-(Dimethylamino)pyridine (DMAP). CPEs showed stable thermal stability up to 190 °C by TGA measurement with a two-step thermal decomposition process. The first decomposition occurred with LiFSI at ~190 °C and the next with the polymer matrix at ~350 °C. CPEs exhibited electrochemical stability of 5.4 V (vs. Li^+^/Li) at 25 °C and a lithium transference number of 0.55. An ionic conductivity of 3.31 × 10^−5^ S cm^−1^ was achieved with CPEs using only PCLA, LiFSI, and DMAP; however, the value increased to 6.62 × 10^−5^ S cm^−1^ at 30 °C when HNTs were added. Since the charged surface of HNTs supports the dissolution of lithium salts, more free ions could be added, and consequently, the increased quantity of mobile charge carriers improved ionic conductivity. With the Li|CPEs|Li symmetric cell, the interfacial stability of CPEs to Li metal could be confirmed. The cell showed a cycling life of 1600 h at a current density of 0.1 mA cm^−2^. In LFP|CPEs|Li, an initial discharge capacity of 161 mAh g^−1^ was obtained at 0.2 C and 60 °C and good electrochemical performance with excellent capacity retention value (92%) after 600 cycles at 1 C [100].

PEGDA, LiTFSI, butyl acrylate (BA), 1-ethyl-3-methylimidazoline bis(trifluoromethylsulfonyl)imide (EMIM TFSI), and diphenyl (2, 4, 6-trimethylbenzoyl) phosphine oxide (TPO) were used for fabricating elastomeric PEL electrolyte (PEL-0.1) enabled by UV induced in situ polymerization [101]. PEL-0.1 had marvelous elongation of 1000% ratio and mild ionic conductivity of 1.19 × 10^−4^ S cm^−1^ at 40 °C. High thermal resistance property was confirmed, where the thermal decomposition of PEL-01 began at an approximate temperature of 330 °C. The electrochemical impedance spectroscopy (EIS) PEL-0.1 was measured at various angles; resistance values at 0°, 45°, 90°, 135°, and 180° twisted angles were 491, 518, 465, 467, and 703 Ω, respectively, which suggests that the developed PEL electrolyte exhibited outstanding flexibility, and the ion conduction was not be greatly impacted during the deformation process. PEL-0.1 with good cycling performance presented capacity retention of 94.3% and ~100% coulombic efficiency after 250 cycles. 

**Figure 8:**
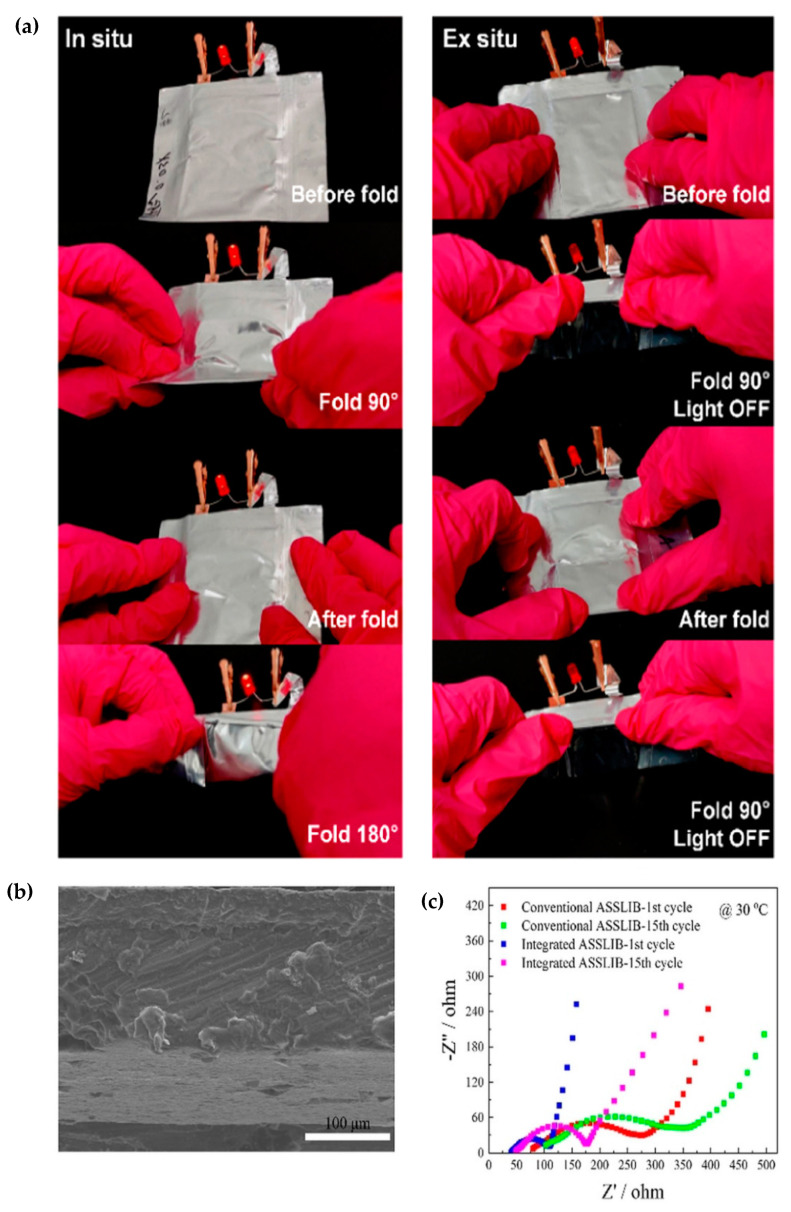
(**a**) Demonstration of the in situ and ex situ pouch-cell-powered LED during folding deformation [102]. Copyright^®^ 2022, American Chemical Society; (**b**) cross-sectional SEM image of interface between electrode and electrolyte; and (**c**) AC impedance of conventional and integrated ASSLIBs after first and fifteen cycle. Reprinted/adapted with permission from Ref. [103]. Copyright^®^ 2020, John Wiley & Sons, Inc.

The solvated ionic liquid-based gel polymer electrolyte (SGPE) is reported, which was prepared by ETPTA, TPO, LiTFSI, and tetraethylene glycol dimethyl ether (TEGDME) [102]. The high thermal decomposition temperature of SGPE was observed at 216 °C, whereas liquid electrolyte-based polymer electrolyte (LGPE) composed of ETPTA and commercial liquid electrolyte showed a low decomposition temperature of 129 °C. A shrinkage test of GPE was conducted at 150 °C for 2 h. Compared with the SGPE without any changes, LGPE experienced significant dimensional reduction with a brunt and broken surface. SGPE exhibited ionic conductivity of 6.30 × 10^−4^ S cm^−1^ at room temperature and was electrochemically stable up to 5.2 V. SGPE also showed uniform lithium deposition property since the overvoltage of SGPE was still approximately 0.04 V after the cycle for 2000 h, indicating small voltage polarization in the symmetric cell. In the LFP half-cell, SGPE showed remarkable cycling performance; the specific capacity of the SGPE cell hardly deteriorated and showed average coulombic efficiency of 99.7% after 750 cycles. Additionally, SGPE showed a capacity retention value of 94.7 and average coulombic efficiency of 99.8% after 450 cycles. As shown in Figure 8a, the LED lamp connected to the pouch cell using PEs with an in situ preparation method emitted light before and after folding, whereas LED using the ex situ pouch cell diminished when folded.

SN-SPEs with ionic conductivity of 4.60 × 10^−4^ S cm^−1^ were fabricated and demonstrated [103]. SN-SPE consisting of ETPTA, SN, HMPP, lithium perchlorate (LiClO_4_), and (ethylene glycol) methyl ether acrylate (mPEGA) exhibited a good elastic tensile performance, which can be stretched above 200%, and good electrochemical stability, up to 4.6 V. The all-solid-state full cells consisting of SN-SPE as an electrolyte, LFP as a cathode, and LTO as an anode were fabricated and measured at 0.2 C. The all-solid-state LIBs (ASSLIBs) enabled by in situ polymerization showed a discharge capacity of 145.93 mAh g^−1^ and good capacity retention of 93.62 % after 100 cycles. However, ASSLIBs fabricated by the ex situ method exhibited a discharge capacity of 98.67 mAh g^−1^ and low capacity retention value of 65.84% after 100 cycles. To investigate interfacial properties between electrode and electrolyte, interface morphology and impedance were measured. The cross-sectional SEM image demonstrated the strong coupling between the cathode and SN-SPE without any discernible delamination (Figure 8b). As shown in Figure 8c, the integrated ASSLIBs had smaller interface resistance than conventional ASSLIBs due to the better interface contact between electrode and electrolyte formed by in situ polymerization [104].

Overall, in situ polymerization using UV photo-initiation had an advantage of fast polymerization time in several tens of seconds and did not affect or damage the inside cell components. Nevertheless, the photo-polymerization can be conducted with the open cell only before the assembly which may cause difficulty of manufacturing regarding the sensitivity of the Li-based electrolytes. Furthermore, the penetration depth of UV light is not guaranteed with the high-density electrode active materials that could restrict the general application of photo-initiation process. Table 6 shows the summary of the polymer electrolytes using UV photo-initiation.

## 5. Conclusions

In this short review, we have summarized and discussed various types and fabrication methods of gel polymer electrolytes (GPEs). Solid polymer electrolytes (SPEs) are composed of a solid polymer matrix that contains an ionic species that can conduct ions. SPEs are typically used in thin film batteries and other applications where the electrolyte must be flexible and conform to the shape of the device. Gel polymer electrolytes (GPEs) are like solid polymer electrolytes, but they are made by suspending the ionic species in a polymer matrix rather than dissolving it in a solvent. This creates a gel-like material that is flexible and can easily conform to the shape of the device. Gel polymer can provide good ionic conductivity as well as mechanical durability and is easy to fabricate. Composite polymer electrolytes (CPEs) are formed by combining a gel polymer electrolyte with inorganic fillers, such as ionic liquid or inorganic nanocomposites, to improve the conductivity of the electrolyte or decrease the crystallinity of the polymer matrix. Composite polymer electrolytes are typically used in high-performance batteries and other electrochemical devices where a high level of conductivity is required.

The promising method of preparing a gel polymer electrolyte is in situ polymerization. A liquid precursor solution is injected into electrodes and then polymerized by thermal- or photo-initiation using a radical initiator. The precursor of liquid phase easily immerses into the internal pores of electrode, and finally, GPEs can be created inside the pores and on the surface of electrode active materials. The GPEs using in situ polymerization can significantly decrease the contact resistance due to the good interfacial contact formed between the electrode and polymeric electrolyte compared with the ex situ preparation method.

In summary, GPEs prepared by in situ polymerization are promising solid phase polymeric electrolytes for Li-ion batteries with increased safety as well as high performance. The in situ polymerized GPEs possess various advantages, including reduced interfacial resistance due to excellent interfacial contact, increased stability of lithium metal-based batteries due to good suppression property of lithium dendrite growth, and the possibility of expansion, such as flexible batteries, owing to good feasibility of PEs. Nevertheless, further studies are needed to improve both electrical and mechanical characteristics of PEs simultaneously to achieve safe and reliable cell performance.

## Figures and Tables

**Table 1:** Comparison of various solid-type electrolytes.

Type of Solid Electrolyte	Advantages	Disadvantages
Oxide	Good thermal stability Good mechanical strength Air stability	Poor processability High interfacial and particle resistance
Sulfide	High ionic conductivity	Sensitive to moisture (H_2_S formation) Poor chemical stability
Polymer	Flexibility Processability Good interfacial properties	Low thermal and mechanical stability

**Table 2:** Comparison of various types of polymer electrolytes.

Polymer Electrolyte	Advantages	Disadvantages
Solid Polymer Electrolytes (SPEs)	Good mechanical properties High thermal stability Solvent-free	Low ionic conductivity at room temperature Non-conformal interface with electrodes.
Gel Polymer Electrolytes (GPEs)	Good ionic conductivity Low volatility and reactivity	Poor mechanical strength
Composite Polymer Electrolytes (CPEs)	Good ionic conductivity High thermal stability Good mechanical properties	Difficult to disperse filler particles
Polymeric Ionic Liquid Electrolytes (PILEs)	High thermal stability Wide electrochemical windows Non-flammability Low vapor pressure	Uncertain ion transport mechanism

**Table 3:** Properties of polymeric host materials.

Polymeric Host	Repeating Unit	Glass Transition Temperature (°C)	Melting Temperature (°C)
PEO	-(CH_2_CH_2_O)_n_-	−64	65
PVDF	-(CH_2_-CF_2_)_n_-	−40	171
PVDF-HFP	-[(CH_2_-CF_2_)-(CF_2_-CF-(CF_3_)]_n_-	−90	135
PAN	-(CH_2_-CH(-CN))_n_-	125	317
PMMA	-(CH_2_C(-CH_3_)(-COOCH_3_))_n_-	105	Amorphous
PVC	-(CH_2_-CHCl)_n_-	80	220
PPC	-[CH(CH_3_)CH_2_OCO_2_]_n_-	35	Amorphous
PDADMACl	-(C_8_H_16_ClN)_n_-	150	-
PVBTMATFSI	-[CH_2_CH(C_6_H_4_CH_2_(CF_3_SO_2_)_2_N)]_n_-	74	-

**Table 4:** Monomers, initiators, and ionic conductivity values of PEs.

Polymer Electrolytes	Monomer	Initiator	Ionic Conductivity (S cm^−1^)	Reference
1.3M LiPF_6_ in EC/DEC = 3/7 (*v*/*v*) with 10 wt.% FEC, ETPTA, BPO, SiO_2_	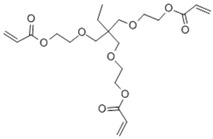	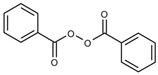	5.20 × 10^−3^	[82]
1.0M LiPF_6_ in EC/DEC = 1/1 (*v*/*v*), PEGDA, AIBN	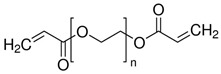	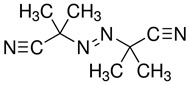	8.81 × 10^−3^	[83]
1.0M LiTFSI in SN, TPPTA, HMPP	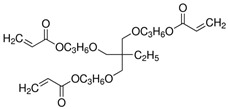	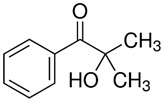	>10^−3^	[84]
1.1M LiPF_6_ in EC/PC/EMC/ DEC = 3/2/3/2 (*v*/*v*/*v*/*v*), DTPTA, BBP	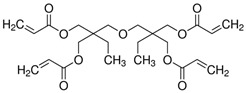	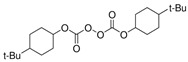	6.20 × 10^−3^	[85]

**Table 5:** Polymer electrolytes using thermal polymerization.

Type of PE	Monomers	Ionic Conductivity (S cm^−1^)	Cell Electrodes	Discharge Capacity (mAh g^−1^)	C-Rate	Reference
GPE	EGPEA	3.35 × 10^−3^	NCM 523/Li	155.0	0.2 C	[91]
GPE	PVFM	8.82 × 10^−3^	LFP/Li	145.0	0.1 C	[92]
GPE	TMPTMA	6.15 × 10^−3^	NCM 811/Li	183.1	0.1 C	[93]
CPE	TEGDA	1.80 × 10^−3^	NCM 111/Li	179.5	0.5 C	[94]
SPE	PEGDA, BA	1.10 × 10^−3^	LFP/Li	93.0	1 C	[95]
GPE	DOL	~10^−4^	LFP/Li	126.5	1 C	[96]
GPE	PEGDMA, PETEA	7.60 × 10^−3^	LFP/Li	~145.0	0.1 C	[88]

**Table 6:** Polymer electrolytes using UV photo-initiation.

Type of PE	Monomers	Ionic Conductivity (S cm^−1^)	Cell Electrodes	Discharge Capacity (mAh g^−1^)	C-Rate	Reference
SPE	ETPTA	2.21 × 10^−5^	LFP/Li	147.0	0.1 C	[97]
SPE	PEGMEMA	2.95 × 10^−5^	LMFP/Li	164.7	0.1 C	[98]
CPE	ETPTA	1.60 × 10^−3^	LCO/Li	152.0	0.2 C	[99]
CPE	PCLA	3.31 × 10^−5^	LFP/Li	155.0	1 C	[100]
GPE	PEGDA, BA	1.19 × 10^−4^	LFP/Li	160.0	0.1 C	[101]
GPE	ETPTA	6.30 × 10^−4^	LFP/Li	141.9	0.5 C	[102]
SPE	ETPTA	4.60 × 10^−4^	LEP/LTO	155.9	0.2 C	[103]

## Data Availability

Not applicable.
